# First Nations Australians’ self-determination in health and alcohol policy development: a Delphi study

**DOI:** 10.1186/s12961-022-00813-6

**Published:** 2022-01-21

**Authors:** Annalee E. Stearne, K. S. Kylie Lee, Steve Allsop, Anthony Shakeshaft, Michael Wright

**Affiliations:** 1 Faculty of Health Sciences, National Drug Research Institute, Curtin University, Perth, Western Australia Australia; 2 Faculty of Health Sciences, enAble Institute, Curtin University, Perth, Western Australia Australia; 3 Faculty of Medicine and Health, Central Clinical School, NHMRC Centre of Research Excellence in Indigenous Health and Alcohol, The University of Sydney, Sydney, New South Wales Australia; 4 The Edith Collins Centre (Translational Research in Alcohol Drugs and Toxicology), Sydney Local Health District, Sydney, New South Wales Australia; 5 Burnet Institute, Melbourne, Victoria Australia; 6 Centre for Alcohol Policy Research, La Trobe University, Melbourne, Victoria Australia; 7 National Drug and Alcohol Research Centre, University of New South Wales, Sydney, New South Wales Australia; 8 Faculty of Health Sciences, Curtin School of Allied Health, Curtin University, Perth, Western Australia Australia

**Keywords:** First Nations Australians, Australia, Self-determination, Policy development, Rights, Alcohol

## Abstract

**Background:**

Recognition of the role of structural, cultural, political and social determinants of health is increasing. A key principle of each of these is self-determination, and according to the United Nations (2007), this is a right of Indigenous Peoples. For First Nations Australians, opportunities to exercise this right appear to be limited. This paper explores First Nations Australian communities’ responses to reducing alcohol-related harms and improving the health and well-being of their communities, with a focus on understanding perceptions and experiences of their self-determination. It is noted that while including First Nations Australians in policies is not in and of itself self-determination, recognition of this right in the processes of developing health and alcohol policies is a critical element. This study aims to identify expert opinion on what is needed for First Nations Australians’ self-determination in the development of health- and alcohol-related policy.

**Methods:**

This study used the Delphi technique to translate an expert panel’s opinions into group consensus. Perspectives were sought from First Nations Australians (*n* = 9) and non-Indigenous Peoples (*n* = 11) with experience in developing, evaluating and/or advocating for alcohol interventions led by First Nations Australians. Using a web-based survey, this study employed three survey rounds to identify and then gain consensus regarding the elements required for First Nations Australians’ self-determination in policy development.

**Results:**

Twenty panellists (*n* = 9 First Nations Australian) participated in at least one of the three surveys. Following the qualitative round 1 survey, six main themes, 60 subthemes and six examples of policy were identified for ranking in round 2. In round 2, consensus was reached with 67% of elements (*n* = 40/60). Elements that did not reach consensus were repeated in round 3, with additional elements (*n* = 5). Overall, consensus was reached on two thirds of elements (66%, *n* = 43/65).

**Conclusions:**

Self-determination is complex, with different meaning in each context. Despite some evidence of self-determination, systemic change in many areas is needed, including in government. This study has identified a starting point, with the identification of elements and structural changes necessary to facilitate First Nations Australian community-led policy development approaches, which are vital to ensuring self-determination.

**Supplementary Information:**

The online version contains supplementary material available at 10.1186/s12961-022-00813-6.

## Background

Recognition of the role of structural, cultural, political and social determinants of health is increasing [1–5], particularly for First Nations Australians^1^ [7, 8]. Despite this, the comparative health and well-being of First Nations Australians is significantly lower than that of other Australians [9–11]. Previous studies have described key elements needed to improve First Nations Australians’ health and well-being; these include recognition and removal of historical and ongoing colonization, dispossession, exclusion and discrimination, and the promotion of First Nations Australian-led decision-making [12–14]. The principle of self-determination, which was identified and recognized by the United Nations (UN) in the years after the Second World War, includes recognition of the right to determine one’s own political status, and to pursue social, economic and cultural development [15]. This is consistent with the collective right of self-determination in the Universal Declaration of Human Rights [16]. However, by construct, Indigenous Peoples were excluded from such rights until the 1960s [17]. Following decades of advocacy, the 2007 UN Declaration on the Rights of Indigenous Peoples (UNDRIP) acknowledged the vital importance of self-determination [18–23]. Australia initially opposed the UNDRIP but became a signatory in 2009, with some caveats^2^ [15, 18, 23–25].

Self-determination is the cornerstone needed to address the historical and ongoing trauma of colonization experienced by Indigenous Peoples, including First Nations Australians [7, 14, 26]. There are also many layers to self-determination, including both personal and community empowerment. While it is complex, challenging to define and means different things to different people in varying contexts [27–30], we define self-determination as “… the internationally recognised and on-going right of Indigenous Peoples to collectively determine their own pathway, within and outside of existing settler societies” [15].

The absence of treaties between Australian governments and First Nations Australians [31] has led to the “operationalization” of self-determination, to some extent, via government policy [32, 33]. This is in contrast to other former British colonies such as Aotearoa-New Zealand, where the Tiriti o Waitangi (Treaty of Waitangi) is a constitutional document [34]. For example, from the 1970s to mid-1990s, self-determination or self-management by First Nations Australians was an Australian Government policy [33]. A key feature of this legislation was the nationally representative Aboriginal and Torres Strait Islander Commission (ATSIC). ATSIC was created as a First Nations Australian-led body of community-elected representatives [35, 36] that was embedded in legislation at a federal level [37]. Its purpose was for First Nations Australians to have input into policy development and funding decisions [38]. ATSIC was disbanded in 2005 despite recommendations for it to continue [39], following a change in Australian Government leadership by then Prime Minister John Howard [32, 40]. Since then, various advisory committees with government-appointed membership have filled some aspects of the roles of ATSIC [41, 42].

First Nations Australian communities have a strong history of leading responses to reduce alcohol-, social- and health-related harms. Examples include supply reduction (e.g. purchasing the hotel/drinking club, and local area controls on availability such as dry areas^3^ and accords^4^) [45, 46], harm reduction (e.g. night patrols,^5^ sobering-up shelters^6^) [47–50], and demand reduction (e.g. campaigns to prevent fetal alcohol spectrum disorder, community-controlled residential treatment) [51–54]. Community ownership and leadership have been identified as integral to the success of these initiatives [55–58].

A critical point is that simply including First Nations Australians in policy development does not equate to self-determination [30, 59, 60]. However, while the right to self-determination in the development of policy, including that related to health and alcohol, affecting First Nations Australians is necessary [60, 61], we were unable to find studies that demonstrated how self-determination could be achieved in this setting [15]. To address this knowledge gap, this study aims to identify expert opinion on what is needed for First Nations Australians’ self-determination in the development of health- and alcohol-related policy.

## Methods

### Study design

The Delphi technique (Delphi) is a multistage iterative survey approach that uses a panel of experts to translate individual opinions into group consensus [62–65]. A key feature is that Delphi allows for diverse perspectives and views [66], which is an essential feature in a study about self-determination, especially where there is a dearth of formal research reports [67]. A series of web-based surveys [65, 68] were used to ensure: participant anonymity [69]; individual perspectives without influence of other panellists [70]; controlled feedback of findings between survey rounds [62]; national contributions without the need for interstate travel [71]; and flexible non-onerous participation to suit each panellist [72–74]. It should be noted that this study was developed within the context of the COVID-19 pandemic, when travel between states/territories in Australia was restricted to only essential travel until November 2020 [71].

#### Formation of the panel

Selection criteria for the panel were as follows: age 18+ years; at least 5 years of professional experience in the health and/or alcohol and other drug (AOD) sectors; and professional involvement in development of policy related to health and alcohol. No definitive number of experts are required for a Delphi study, with variation based on the scope of the study and available resources [62, 75]. We aimed to recruit a diverse panel [76] in relation to gender, indigeneity, region (Australia-wide; remote through to urban contexts) and related professional experience and qualifications (clinical, research, policy, advocacy). Perspectives were also sought from non-Indigenous peoples with experience in developing, evaluating and/or advocating for alcohol interventions led by First Nations Australians.

#### Panel recruitment

All panellists were recruited using purposive sampling. Thirty-nine experts (68% First Nations Australian) were invited to participate by personalized email or phone call (AES). Of these, 31 experts had professional connections with the research team (AES, KL, MW, SA). The remainder (*n* = 8) were suggested for recruitment by other panellists. Even though objectivity is important, research with First Nations Australian communities requires interaction and accountability between the researchers and participants [73, 77, 78].

### Ethics

Ethical approval was provided by the Curtin University Human Research Ethics Committee (HRE2019-0729) and the Central Australian Human Research Ethics Committee (CA-19-3525). Participation was opt-in and voluntary. Informed consent was sought electronically prior to the commencement of each survey.

### Procedure

#### Data collection

Data were collected (by AES) using an electronic survey across three sequential rounds in September, October and December 2020. Inspired by the classic Delphi approach [62, 76], the purpose of round 1 was to gain panellists’ views and perspectives primarily via open-ended qualitative questions. Rounds 2 and 3 used structured questions, with open-text fields for panellists to expand on responses (Table 1). When appropriate, continuous (*n* = 10) or categorical (*n* = 3) Likert scales were used for ranking of response options [79].

**Table 1:** Summary of surveys by round

Questions	Response type	Number of questions (elements)^a^
Round 1^a^		
A. Demographics: experience, qualifications, jurisdictions^b^		
B. Essential elements needed for policy development processes to be self-determinative	Open-ended text field	11
C. The degree of self-determination evident in evidenced-based examples of the policy development process	10-point Likert scale^c^ Open-ended text field	8
D. Identifying the stages when it is essential for First Nations Australians to be included in policy development and suggested examples of First Nations Australians’ self-determination in policy development processes	2 categorical questions^d^ Open-ended text field	15
Round 2^a^		
Q1. Support for these existing elements and changes to others, would enable First Nations Australians’ self-determination to be recognized	4-point Likert scale^e^ Open-ended text field	(6)
Q2. There were a number of values identified that should underpin policy development processes for it to be seen as self-determination	4-point Likert scale^e^ Open-ended text field	(8)
Q3. Self-determination in alcohol policy requires the policy-makers to use processes that ensure First Nations Australian/s…	7-point Likert scale^f^ Open-ended text field	(16)
Q4. Self-determination in alcohol policy development requires decision-making processes that…	7-point Likert scale^f^ Open-ended text field	(10)
Q5. Self-determination in alcohol policy development requires that First Nations Australians are involved in the process with representation from First Nations Australians…	7-point Likert scale^f^ Open-ended text field	(12)
Q6. At implementation, alcohol policy should include approaches that ensure it…	7-point Likert scale^f^ Open-ended text field	(8)
Q7. Examples	7-point Likert scale^f^ Open-ended text field	(6)
Round 3^a^		
Q1. Support for these existing elements and changes to others, would enable First Nations Australians' self-determination to be recognized	7-point Likert scale^f^ Open-ended text field	(1)
Q3. Self-determination in alcohol policy requires the policy-makers to use processes that ensure First Nations Australian/s…	7-point Likert scale^f^ Open-ended text field	(4 + 2)
Q4. Self-determination in alcohol policy development requires decision-making processes that…	7-point Likert scale^f^ Open-ended text field	(3 + 3)
Q5. Self-determination in alcohol policy development requires that First Nations Australians are involved in the process with representation from First Nations Australians…	3-point Likert scale^g^ Open-ended text field	(10)
Q6. At implementation, alcohol policy should include approaches that ensure it…	7-point Likert scale^f^ Open-ended text field	(2)
Q7. Examples	4 options^h^ Open-ended text field	(6)

^a^Round 1—the number of questions for the section; round 2 and round 3—the number of elements (subthemes) for each question, arising from analysis of round 1 and round 2 survey data

^b^See Table 2

^c^1–10 continuous Likert scale: 1 = not self-determination to 10 = definitely self-determination

^d^Eight options (select all that apply): not at all; agenda-setting stage; consultation; policy creation; implementation; monitoring; evaluation; all stages

^e^Four-option Likert (select one): non-negotiable and can be implemented now; non-negotiable, but is aspirational and unlikely at present; ideal but not necessary; not self-determination

^f^1–7 continuous Likert scale: 1 = not self-determination to 7 = non-negotiable necessary for self-determination

^g^Three-option Likert (select one): always include; include in some contexts but not all; inclusion is not self-determination

^h^Four options (select all that apply): type of representation; stage that First Nations Australians were involved; how First Nations Australians were involved; and aim of the example provided

Each survey was tested prior to distribution for usability and timeliness by members of the research team (AES, SA, MW, KL) and by First Nations and non-Indigenous Australians not involved in the study (*n* = 6). After survey finalization, a personal survey link was sent to each participant (by AES). Responses were analysed after each round, and a plain English summary was then emailed to panellists, along with the next survey. Survey links were active for 3 weeks, with up to four personalized reminders given, usually by AES. Panellists were also given the opportunity to complete the surveys by videoconference or phone interview (with AES). At the completion of round 3, panellists received a gift card to acknowledge their contribution to the study.

#### Round 1: survey

In round 1, the survey consisted of four sections (Table 1): (a) demographics (e.g. professional experience, qualifications, jurisdictions); (b) essential elements needed for the policy development processes to be described as involving self-determination; (c) degree of self-determination in examples of policy development processes, and the type of representation and methods needed to be inclusive of First Nations Australians; and (d) essential stages for First Nations Australians to be included in policy development and suggested examples.

#### Round 2: survey

In round 2, the survey aimed to seek consensus on seven questions, derived from round 1 analysis (Table 1). Q1–Q2: macro-level^7^ conditions and values necessary to achieve self-determination in policy. Q3–Q6: elements needed to enable self-determination in policy development processes; (Q3) macro-level conditions necessary for self-determination in policy development; (Q4) elements in decision-making processes; (Q5) types of representation; and (Q6) elements needed in policy implementation. As ranking of representation types (round 1C) did not achieve consensus, and panellists suggested other response options, these were integrated into Q5 in round 2. Q 7: Brief real-life vignettes were provided to show how Australian policy has been developed with First Nations Australians (suggested by panellists; prepared by AES). Two de-identified vignettes were examples of First Nations Australian community-specific alcohol harm minimization programs. The remaining vignettes were national examples of First Nations Australians being included in policy processes. Vignettes were ranked on perceived self-determination in policy development.

#### Round 3: survey

In round 3, we sought consensus on six questions and related elements that did not reach consensus in round 2. Questions focused on the following: (Q1) structural changes at a federal government level deemed necessary for First Nations Australians’ self-determination in policy development processes; (Q3) essentials for self-determination to be part of policy development processes; (Q4) types of representation needed; and (Q6) implementation. Round 3 also included elements suggested by panellists (related to Q3 and Q4). Q5 and Q7 were asked again (from round 2), with response categories amended based on panellists’ feedback.

### Data analysis

All survey data were collected using Qualtrics [80], a web-based survey platform. Qualitative data were analysed (by AES) using content analysis [81]. Text-based responses were reviewed and thematically analysed [82]. Coding was reviewed by another author (KL). Responses were grouped into similar themes, which became round 2 questions, with the subthemes being elements that were ranked within each question. Additional checking from a third author (MW) ensured that data were appropriately categorized.

Consensus level was set at 80% agreement in panellists’ rankings [76, 83]. In rounds 2 and 3, the seven-point continuous Likert scales were collapsed into three categories (1–2: not self-determination; 3–5: possibly; 6–7: definitely self-determination). In round 2, the categorical responses for Q1 and Q2 were collapsed into three groups (1: non-negotiable and can be implemented now, and non-negotiable, but is aspirational and unlikely at present; 2: ideal but not necessary; and 3: not self-determination).

## Results

### Panel of experts

Twenty individuals (45% First Nations Australian) from six Australian states or territories^8^ participated in at least one survey round. The majority of panellists (95%) completed two or more survey rounds, with 60% completing all three rounds (Table 2). Despite reaching a First Nations Australian majority prior to commencement, four experts withdrew and did not participate in any surveys. The time of the year and competing priorities (including increased work responsibilities related to COVID-19) were the main reasons reported by panellists for survey non-completion. One panellist preferred to complete rounds 2 and 3 via phone. Just over half (*n* = 11/20) of the panellists (*n* = 3 First Nations Australians) were in academic roles, with more than 200 years of combined experience. The remaining panellists were either executive officers (*n* = 5) or senior program/area managers (*n* = 4), with more than 170 years of combined professional experience in First Nations Australian community-led organizations and more than 100 years of experience on national or state advisory committees (health and AOD).

**Table 2:** Expert panel in a study of self-determination in policy development processes (*n* = 20)

	First Nations Australian	Non-Indigenous^a^	Total
Panellists			
Unique individuals across entire Delphi	9	11	20
Survey 1	7	10	17
Survey 2	8	10	18
Survey 3	7	10	17
Jurisdictions of interest			
National	3	1	4
New South Wales	3	1	4
Northern Territory	3	10	13
Queensland	3	3	6
South Australia	2	1	3
Victoria	2	–	2
Western Australia	1	3	4
Highest completed qualifications			
Associate/graduate degree	2	–	2
Master or doctorate	5	11	16
Vocational/TAFE diploma	2	–	2
Professional experience: number of individuals (total years of experience)			
Academic: university/research	4 (40)	7 (176)	11 (216)
Government: federal/state/territory	3 (46)	3 (17)	6 (63)
Community-led organization: First Nations Australian	8 (130)	5 (96)	13 (226)
Peak body: First Nations Australian	6 (68)	3 (30)	9 (98)
Advisory committees: national/state/territory	9 (118)	5 (67)	14 (185)
Other non-government/not-for-profit organization	2 (18)	2 (22)	4 (40)
Primary healthcare/medical service	7 (74)	4 (117)	11 (191)
Areas of specialization			
Alcohol	8	11	19
Clinical/medical	4	2	6
Community-led advocacy	3	5	8
Health	8	10	18
Social policy	5	5	10
Workforce training (health and alcohol)	6	1	7

^a^ Indigeneity not declared (*n* = 1)

### Round 1

Seventeen panellists completed the round 1 survey. Six main themes (questions) (Table 1) and 60 related subthemes (elements) (Table 3) were identified. The themes were multilayered, recognizing that changes at a macro (federal government) through to micro 9 level were needed to develop and implement policy with First Nations Australian communities. Panellists identified that First Nations Australians’ self-determination in policy development requires considerations in the following areas: (1) support from the federal government at a macro level; (2) values underpinning the entire process; (3) specific elements essential to the entire policy process; (4) decision-making within the policy development process; (5) First Nations Australian representation; and (6) essential elements for implementation. In addition, panellists suggested 10 examples of First Nations Australians’ self-determination in policy development processes, six of which were included as real-life vignettes (Q7).

**Table 3:** Consensus ranking of elements needed for self-determination in policy development

Themes—subthemes
Q1 Support for these existing elements and changes to others would enable First Nations Australians’ self-determination to be recognized
80–100% support
Recognition and support for the role of Aboriginal community-controlled organizations is needed to ensure there is a First Nations Australia voice
Recognition that the First Nations Australian world view and collective identity is different from that of non-Indigenous Australians is needed throughout all processes
Constitutional recognition of First Nations Australians and a collectively decided voice to parliament are needed
Democratic processes embedded throughout the policy development system are needed
Treaty/ies between First Nations Australians and the state and Australian governments that recognize the sovereignty of First Nations Australians are needed
Change across the wider government and policy systems is needed to address and remove the structural determinants of health^a^
Q2 There were a number of values identified that should underpin policy development processes for it to be seen as self-determination
80–100% support
The human rights of First Nations Australians are meaningfully considered
The human rights of First Nations Australians are protected
First Nations Australian culture and decision-making processes (consensus) are evident throughout the process
The process is informed by the priorities and needs of First Nations Australian community/ies that are affected/impacted
The diversity of First Nations Australians is recognized and accepted
There is improvement of First Nations Australian individuals’ and communities’ lives
The process is driven and directed by First Nations Australian leadership and governance
First Nations Australians have significant influence and power over the process
Q3 Self-determination in alcohol policy requires the policy-makers to use processes that ensure First Nations Australian/s…
80–100% support
Are given adequate time for decision-making
Receive feedback promptly and in a suitable format
Are involved in the codesign/co-development of policy
Are consulted early in the policy-making process
Have the opportunity to contribute to the policy-making process
Are involved in parts of the policy-making process^a^
Communities are able to hold the policy-makers accountable
Policy-makers can develop and build trust throughout
Are involved throughout the policy-making process
Are resourced and funded to be included at all stages
Two-way sharing (decision-making power and being informed of what has worked elsewhere)^a,b^
Are involved in evaluating the policy
Are involved in monitoring the policy
Local culture and language/s are considered and adjusted for in the policy-making process
Less than 80% support
Are involved in ALL data processes relating to alcohol policy (data sovereignty)^b^
Community/ies have autonomy in the policy-making process
Communities define the policy-making process
Communities can control the policy-making process throughout
Q4 Self-determination in alcohol policy development requires decision-making processes that…
80–100% support
Are participatory and transparent for all parties
Involve First Nations Australians
Are evaluated and monitored, with prompt response to feedback
Recognize the cultural obligations and expectations of First Nations Australians
Are adapted for local context
Are led by First Nations Australians
Are defined by First Nations Australians
Less than 80% support
Are not circumvented or changed at higher tiers of government^b^
Are democratic
Are balanced between the evidence base and community preferences^b^
Are consensus-based
Give First Nations Australian communities/participants veto power at all levels
Give First Nations Australian community-controlled organizations collective veto power at all levels^b^
Q6 At implementation, alcohol policy should include approaches that ensures it…
80–100% support
Is evaluated and monitored, with prompt response to feedback
Is not discriminatory against First Nations Australians’ human rights
Is respectful of the priorities of First Nations Australians and their communities
Involves First Nations Australians in the implementation decision-making
Results in the changes desired by the affected community/ies
Involves First Nations Australians in the resource allocation decision-making
Less than 80% support
Supports First Nations Australian leading service provision
Is translatable across the wider government and policy systems

^a^> 80% consensus was reached in survey 3

^b^Element only in survey 3

**Fig. 1:**
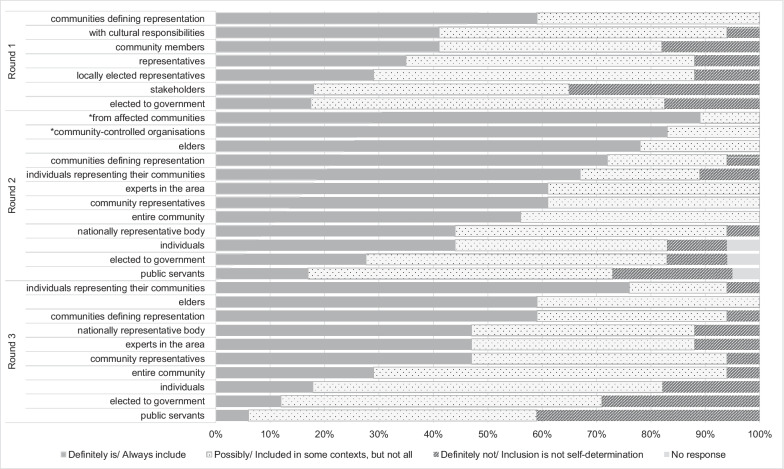
Ranking of First Nations Australian representation in policy development

*Types of First Nations Australian representation in policy processes.* No elements reached more than 80% agreement when ranking types of representation (Fig. 1). One element (communities defining representation) was ranked by all panellists as being “definitely” (59%) or “possibly” (41%) self-determination. Of the remaining elements, involvement as stakeholders was ranked by just over one third (35%) of panellists as “definitely not self-determination”.

*Ways of including First Nations Australians in policy processes.* Ways that First Nations Australians can be included in policy development processes did not reach consensus (Table 4). Three elements were ranked by all panellists as being “possibly or definitely self-determination”: First Nations Australian-defined approach; First Nations Australian-defined representative body/group; and First Nations Australian-led lobbying. At the other end of the scale, two elements were ranked as “not self-determination”: general consultation (35%) and policy that is developed via a specific representative body (24%). Given this lack of consensus, it was clear that the elements being ranked were specific to particular processes, and many were already integrated in the round 2 questions; thus this question was excluded from subsequent rounds.

**Table 4:** Ways of including First Nations Australians in policy development (*n* = 17) (round 1)

	Self-determination	Possibly	Not self-determination
Advisory group/committees	18	71	6
Community consultation activities	24	53	18
Focus groups/meetings/working groups/workshops	12	65	18
General consultation—interviews/questionnaires/submission	6	53	35
First Nations Australian-defined approach	65	29	–
First Nations Australian-defined representative body/group	47	47	–
First Nations Australian-led lobbying	41	53	–
Nomination/voting a number of First Nations Australians to representative body	24	59	12
Nomination/voting for a First Nations Australian representative body (individual)	24	59	12
Policy meetings, roundtables, drafting policy	12	71	12
Via specific representative body/group	18	53	24

**Fig. 2:**
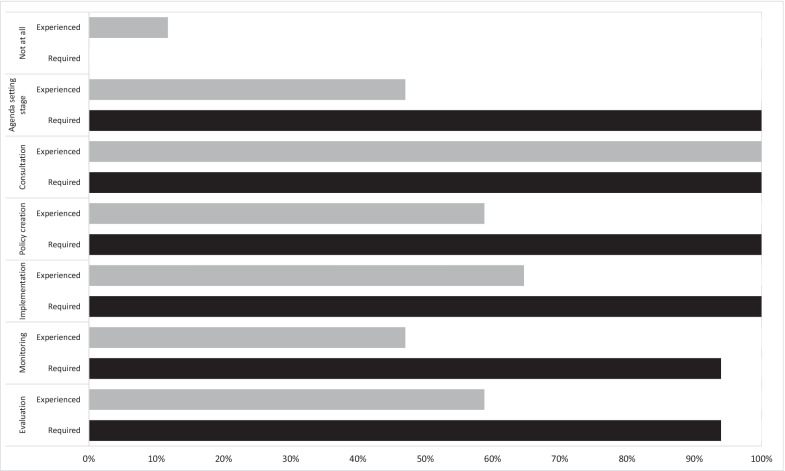
Stages of First Nations Australians’ inclusion in policy development

*Stages of First Nations Australian inclusion in policy processes.* All panellists had “directly seen or been involved in” policy processes where First Nations Australians were included through “consultation” (Fig. 2). Just four in 10 panellists (*n* = 7/17, 41%) had directly seen or been involved in processes that included First Nations Australians at all stages (agenda-setting through to evaluation). Panellists had witnessed or been involved in policy processes that included First Nations Australians in 47–65% of the remaining stages (Fig. 2). Two panellists had seen or been involved in policy processes that had not included First Nations Australians at all. All but one panellist (94%) said that First Nations Australians should be included in all stages of the policy development process. The one dissenting panellist asserted that monitoring and evaluation of policy should be independent.

### Rounds 2 and 3

In round 2, panellists agreed on more than two thirds of the elements needed for First Nations Australians’ self-determination to be evidenced in policy development processes (Q1–Q6: *n* = 40/60, 67%; Table 3). These are detailed below. An additional five elements were suggested for ranking in round 3. In round 3, an additional three elements reached consensus (Q1–6: *n* = 43/65, 66%). Excluding Q5, between 54 and 100% of elements reached consensus in each question. Little agreement was reached on the considerations for self-determination in the real-life vignettes provided (Q7).

*Q1 and Q2: Macro-level conditions and values needed in the policy development process.* In round 2, there was almost universal agreement in the ranking of both the macro-level conditions necessary for self-determination (Q1: *n* = 5/6, 83%) and the underlying values that should be in place (Q2: *n* = 8/8, 100%) in policy development processes (Table 3). One element—recognition and support for the role of Aboriginal community-controlled organizations to ensure a First Nations Australian voice—was ranked “non-negotiable and can be implemented now” by 89% of panellists. All other statements in Q1 and Q2 were ranked by the majority of panellists (83–94%) as either “non-negotiable and can be implemented now” or “non-negotiable, but is aspirational” (Table 1). Despite not reaching consensus in round 2, nearly nine in 10 panellists (*n* = 15/17, 88%) agreed in round 3 that “change across the wider government and policy systems to address and remove the structural determinants of health” is required to ensure First Nations Australians’ self-determination in policy development processes. The detailed results are available [see Additional files 1, 2 and 3].

*Q3: Essentials in the process of developing policy.* The majority of essential elements necessary for self-determination in the process of developing policy reached agreement (Table 3). In round 2, three quarters of elements (*n* = 12/16, 75%) reached consensus and an additional two elements were proposed (two-way sharing and data sovereignty). In round 3, consensus was achieved in the ranking of nearly eight in 10 elements (*n* = 14/18, 78%). Of these, all were ranked as “definitely needed for self-determination”. Two elements had 100% agreement: “receive feedback promptly and in a suitable format” and “given adequate time for decision-making”. In round 3, elements that did not reach consensus were all ranked as “definitely needed” by half to three quarters of panellists (53–76%). The detailed results are available [see Additional files 1 and 4].

*Q4: Decision-making processes in policy processes.* In round 2, consensus was reached for seven out of 10 (70%) elements regarding the nature of decision-making in policy development to ensure First Nations Australians’ self-determination (Table 3). In round 2, an additional three elements were suggested: (1) are not circumvented or changed at higher tiers^10^ of government; (2) are balanced between the evidence base and community preferences; and (3) give First Nations Australian community-controlled organizations collective veto power at all levels.

All elements that reached consensus were ranked as “definitely necessary for self-determination”. Total agreement (100%) was reached for two elements (participatory and transparent for all parties; involves First Nations Australians). In round 3, no further agreement was reached for the remaining six elements (*n* = 7/13, 56%; Table 3). Elements that did not reach consensus were ranked as “definitely needed” by just under half to three quarters of panellists (round 3: 47–76%). The detailed results are available [see Additional files 1 and 5].

*Q5: Representation by First Nations Australians in policy processes.* As in round 1, minimal consensus was achieved in relation to the types of First Nations Australian representation that is necessary for self-determination in policy development. In round 2, just two items reached consensus (*n* = 2/12, 16%; Fig. 1). Panellists agreed on two types of First Nations Australian representation (i.e. to include individuals from affected/impacted communities, 89%; and locally representative/community-controlled organizations, 83%). No further consensus was reached in round 3. As presented in Fig. 1, there were three elements (round 1: stakeholders; round 2: public servants; round 3: public servants and elected government officials) where the combined rankings of “definitely” and “possibly” self-determination did not achieve consensus (59–73%). In round 3, four in 10 (41%) panellists ranked public servants’ inclusion as “not self-determination”.

*Q6: Factors essential in the implementation of policy.* In round 2, panellists agreed on three quarters of the elements that were seen as being necessary in the process of policy implementation (*n* = 6/8, 75%; Table 3). Total agreement was achieved for three elements that were “definitely needed for self-determination” (i.e. evaluated and monitored with prompt response to feedback; not discriminatory against First Nations Australians’ human rights; and respectful of the priorities of First Nations Australians and their communities). In round 3, the remaining elements (*n* = 2) had similar rankings to round 2 but did not exceed 76% agreement. The detailed results are available [see Additional files 1 and 6].

*Q7: Real-life vignettes of First Nations Australian involvement in policy development processes.* In round 2, panellists ranked the degree of self-determination they believed was evident in six real-life vignettes (Table 5). Consensus was achieved in one example, community-led restrictions on takeaway alcohol in Fitzroy Crossing [85, 86]. In round 3, panellists considered which factors were important when considering evidence of self-determination in the vignettes provided. In three examples, consensus was reached with one element—representation of First Nations Australians in the policy development process (ranked 69–94% across the examples; Table 5). Consensus was not reached for the other elements: the stage that First Nations Australians were involved in (31–69%); how First Nations Australians were involved (44–63%); and the aim of the policy (19–38%).

**Table 5:** Ranking of examples of First Nations Australians’ involvement in policy development (Q7)

	Round 2				Round 3			
	Definitely not (%)	Possibly (%)	Definitely is (%)	Representation in the process (%)		Stage involved (%)	How involved (%)	Aim (%)
Aboriginal and Torres Strait Islander Commission	28	39	33	81		31	50	25
National Indigenous Drug and Alcohol Committee	6	44	50	75		38	56	19
*Uluru Statement from the Heart*	6	22	72	94		63	63	38
Aboriginal community-controlled organizations	–	22	78	81		69	56	31
Example #1—Fitzroy Crossing restrictions	–	17	83	69		56	44	31
Example #2—Groote Eylandt permit system	6	17	78	69		56	44	31

## Discussion

To our knowledge, this is the first study to explore what is necessary for First Nations Australians to achieve self-determination in the development of health- and alcohol-related policy. While self-determination is recognized as important to improve health and well-being [87–89], how First Nations Australians have been supported to action it in health and alcohol policy development is limited [32, 60, 90]. The expert panellists identified a series of complex, interrelated and interactive elements that would be needed to scaffold First Nations Australians’ self-determination in policy development processes. Three factors warrant consideration: (1) elements that would help to enable self-determination in policy development do not exist in isolation; (2) community-first or “ground-up” approaches to policy development are integral; (3) the impact of the current Australian policy context (e.g. geopolitical factors) in which policies on health and alcohol would sit.

### Interrelated nature of elements needed for self-determination to be evident in policy development processes

Panellists agreed that First Nations Australians need to be involved in all stages of the policy process for self-determination to be possible (i.e. agenda-setting, consultation, policy creation, implementation, monitoring and evaluation). The lack of consensus achieved when panellists were asked to rate six “real-life” examples (Table 5) reflects Larsen’s [91] findings that self-determination in policy development may not be present across all stages. For example, it is possible for self-determination to be evident in some stages of the policy process and completely absent in others [15, 91]. Further to this, the type of representation of First Nations Australians (Fig. 1) needs careful consideration. These results indicate and support recent pleas for representation beyond experts, individuals and “blanket” representation, as these are not self-determination or appropriate [15, 59].

Representation was seen as involving First Nations Australians in all stages of policy development by all but one panellist. The one dissenting panellist explained that monitoring and evaluation should be conducted independently (i.e. with no assumption that it be conducted by First Nations Australians). While there is a need for independence in the monitoring and evaluation of policy, the Productivity Commission report (2020) positions the role of First Nations Australians at the epicentre when evaluating policy that affects them and their communities [92]. It is clear that First Nations Australians must be involved throughout the development of policy, but representation remains contentious, as the views are as diverse as the communities and Peoples involved.

### “Ground-up” policy approach

Panellists agreed that policy processes should be led and defined by First Nations Australians from the “ground-up”. However, panellists suggested that this can only be achieved when community priorities and voices are placed first [28, 58, 93]. For this to happen, relationships with First Nations Australian communities need to be prioritized and their diversity recognized [7]. Panellists agreed that with meaningful community engagement and involvement throughout the policy development process, community ownership can be created [28, 32], as well as a policy that is directly relevant to the affected community [93–95].

### The impact of the current Australian health and alcohol policy context on achieving self-determination

While all panellists acknowledged the right to self-determination, some saw it as a “right” irrespective of the current policy context. In contrast, other panellists took a pragmatic approach and saw self-determination as an aspiration in the current Australian geopolitical landscape. Nonetheless, panellists agreed that structural change [96] was required for self-determination to have a better chance at success. For example, the Australian government recently endorsed and supported a regionalized consultation process to be undertaken to recognize First Nations Australians in the Australian Constitution. Presented with the outcome of this consultative process in May 2017—the *Uluru Statement from the Heart*—the prime ministers have since vetoed the request for constitutional recognition of First Nations Australian voices in parliament [97, 98], opting instead for legislative-based rights [99].

Another geopolitical issue worthy of consideration is how alcohol-related policy is contextualized, in contrast to other types of health-related policy [57]. In Australia, efforts to develop alcohol-related policy have been underpinned by protectionism [100], community safety [101], justice and criminalization [102, 103]. This approach dismisses the historical and health context of alcohol consumption by First Nations Australians [57]. It also undermines the valuable perspectives of First Nations Australian community-controlled health organizations in the development of alcohol-related policy. First Nations Australian community-controlled organizations have grown from a rich history of self-determination [104, 105]. From an individual community level through to regional and state/territory umbrella affiliates, community-controlled organizations have long-standing systems in place to represent their local communities. This would contribute unique insights to developing alcohol-related policy within a health context [27, 32, 45, 57]. To ensure diversity of First Nations Australian representation, community-controlled health organizations should be included as one source, alongside a spectrum of other types (or groups) of First Nations Australian representation [27, 32].

### Limitations

This study has a number of limitations that need to be considered. The lack of randomness in recruitment is often cited as a major criticism of Delphi studies [62, 106], as the panel of experts is selected by the research team. However, targeted recruitment of panellists with extensive knowledge and experience in a specific area of study has been shown to be a key strength of Delphi studies [75]. In this study, care was taken to assemble a panel with specific knowledge and expertise. The panel’s rich experience as leaders in their respective fields provided an evidenced-based opinion from which consensus was sought. Panellists with limited technology access or comfort may have preferred a phone or face-to-face interview rather than an online survey (*n* = 1 panellist chose to complete phone surveys). A varied response rate (85–90%) was achieved across the three rounds due to panellists’ professional commitments and other priorities (including *n* = 9/20 who were involved in or led local COVID-19 responses). The existing relationships between the research team and panellists may also be seen as a potential source of bias. The qualitative approach used in round 1 assisted in mitigating this, as panellists presented a diverse range of views and perspectives and were not responding to the views of the research team. While during 2020 Australia managed to control the spread of COVID-19, the timing of this study (September–December 2020) may have influenced the choices made by panellists [71]. The focus placed on self-determination added complexity to the study, particularly during analysis. Most Delphi studies use discrete categorical responses [107]. However, this study sought to preserve the varying shades of what constitutes self-determination and the panellists’ right to cast their vote on survey questions using a continuous ranking scale [108].

## Conclusion

Systemic change is needed for self-determination by First Nations Australians to be evident in the development of health and alcohol policy. Changes are necessary at each level of government, as well as in the process of developing policy, in order for First Nations Australians’ self-determination. The diversity and polarity of panellists’ views in this study highlight the complexities in defining self-determination, especially within the health and alcohol policy development context. Closer examination of specific policies locally is needed to assess the level of self-determination that First Nations Australians have in the development of health- and alcohol-related policies. While efficient for policy-makers, policy development processes led by policy-makers was seen by panellists as not self-determination. As long as the processes are defined by the government, First Nations Australians will not have self-determination. Recognition of First Nations Australians’ right to—not just a principle of—self-determination is vital to improve their health and well-being. This recognition, along with community-led approaches, and embedding of this right within state and federal government constitutions are key.

## Supplementary Information

**Additional file 1: Table S1.** Percentage of each ranking for Q1–Q6a (rounds 2 and 3). Provides the detailed survey results for rounds 2 and 3, for Q1 to Q6 (exc. Q5).

**Additional file 2: Figure S1.** Ranking of macro-level elements to facilitate First Nations Australians' self-determination (Q1). Presents the rankings by proportion for all responses in Q1 for rounds 2 and 3.

**Additional file 3: Figure S2.** Ranking of values that should facilitate First Nations Australians' self-determination (Q2). Presents the rankings by proportion for all responses in Q2 for rounds 2 and 3.

**Additional file 4: Figure S3.** Ranking of the policy processes needed to ensure self-determination is evident (Q3). Presents the rankings by proportion for all responses in Q3 for rounds 2 and 3.

**Additional file 5: Figure S4.** Decision-making processes needed policy development for self-determination (Q4). Presents the rankings by proportion for all responses in Q4 for rounds 2 and 3.

**Additional file 6: Figure S5.** Factors necessary for self-determination in the implementation (Q6). Presents the rankings by proportion for all responses in Q6 for rounds 2 and 3.

## Data Availability

Data sharing is not applicable to this article as no datasets were generated or analysed during the current study.

## Abbreviations

ATSIC: Aboriginal and Torres Strait Islander Commission
COVID-19: Coronavirus disease 2019, caused by severe acute respiratory syndrome coronavirus 2 (SARS-CoV-2)
Delphi: Delphi technique
Q1–Q7: Question 1 through to question 7
UNDRIP: United Nations Declaration on the Rights of Indigenous Peoples

## Footnotes

1 “First Nations Australians” has been used to collectively refer to all the Peoples within Australia also known as: “Indigenous”, “Aboriginal” and “Torres Strait Islander”. This phrase has been used with consideration and recognition of the diversity of nations, peoples and cultures that continue to live and care for the lands now referred to as Australia, the islands of the Torres Strait and the waters surrounding them [6].

2 Australia (Canada, New Zealand, and the United States of America) objected to the UNDRIP on the grounds of two articles: requiring Indigenous Peoples’ consent in the development of policy (Article 19) and use of land and resources (Article 32) [15, 18, 23–25].

3 “Dry areas” are areas where the public consumption, and sometimes possession, of alcohol is not permitted [43, 44].

4 “Accords” are locally negotiated agreements between the retailers and community, regarding the sale and availability of alcohol [43].

5 “Night patrols” are First Nations Australian community-initiated groups that travel around the local community with the primary purpose of reducing alcohol-related social harms (e.g. public intoxication, interpersonal violence) [47, 48].

6 “Sobering-up shelters” are facilities where intoxicated individuals are diverted from police lockups and public spaces to a safe place where they can recover. While they may be encouraged to seek further assistance once sober, the primary purpose is to focus on immediate safety [49, 50].

7 “Macro”: These are elements or levels that are large or broad in scope. Within this context, these are overarching approaches by the Australian Government, which may not immediately or directly affect First Nations Australians’ lives.

8 The Commonwealth of Australia is made up of six states and two mainland territories (Australian Capital Territory and the Northern Territory). The mainland territories have the same status as the states, except the Australian Government can amend and rescind any legislation enacted by the Territory governments [84].

9 “Micro”: These are smaller elements that are more tangible, and ideally evident in each policy development process.

10 Within the Australian context, in addition to the Australian federal government, there are another two levels of formal government. Each state and mainland territory has a government; then within each of these are local government councils. Each level of government has different responsibilities in the development of health- and alcohol-related policy (84).
