# Supplementary material for: First Nations Australians’ self-determination in health and alcohol policy development: a Delphi study
Source: Health Res Policy Syst. 2022 Jan 21;20:12. doi: 10.1186/s12961-022-00813-6 (PMC8777453; doi:10.1186/s12961-022-00813-6)
Supplement: Supplementary file 1 — Additional file 1: Table S1. Percentage of each ranking for Q1–Q6a (rounds 2 and 3). Provides the detailed survey results for rounds 2 and 3, for Q1 to Q6 (exc. Q5). [file 12961_2022_813_MOESM1_ESM.docx]

Additional table 1: Percentage of each ranking for Questions 1–6^a^ (Rounds 2 and 3)

|  | | | **Ideal but not necessary** | **Non-negotiable** | | | | **Not self-determination** | | **No response** |  |
| --- | --- | --- | --- | --- | --- | --- | --- | --- | --- | --- | --- |
|  |  |  |  | **& can be implemented now** | | **but is aspirational & unlikely at present** | |  |  |  |  |
| *Q1 Self-determination is not only a right to be recognised at the policy development level. Support for these existing elements and changes to others, would enable First Nations Australians' self-determination to be recognised.* | | | | | | | | | | | |
| Round 2 | | | | | | | | | | | |
| Constitutional recognition of First Nations Australians, and a collectively decided voice to parliament are needed | | | 11 | **50** | | **33** | | - | | 6 |  |
| Democratic processes embedded throughout the policy development system are needed | | | - | **61** | | **22** | | 11 | | 6 |  |
| Treaty/ies between First Nations Australians and the state and Australian governments that recognise the sovereignty of First Nations Australians are needed | | | 11 | **61** | | **22** | | - | | 6 |  |
| Recognition and support for the role of Aboriginal Community Controlled organisations is needed to ensure there is a First Nations Australia voice | | | - | **89** | | 6 | | - | | 6 |  |
| Recognition of the First Nations Australian worldview | | | - | **72** | | **17** | | 6 | | 6 |  |
| Change across the wider government and policy systems is needed to address and remove the structural determinants of health | | | - | 61 | | 17 | | 17 | | 6 |  |
| Round 3 | | | | | | | | | | | |
|  | | | 1–2^b^ | | 3–5^c^ | | 6–7^d^ | | No Response | | |
| Change across the wider government and policy systems is needed to address and remove the structural determinants of health | | | - | | 12 | | **88** | | - | | |
| Round 2 | | | | | | | | | | | |
|  | | | **Ideal but not necessary** | **Non-negotiable** | | | | **Not self-determination** | | **No response** |  |
|  |  |  |  | **& can be implemented now** | | **but is aspirational & unlikely at present** | |  |  |  |  |
| *Q2 There were a number of values identified that should underpin policy development processes for it to be seen as self-determination.* | | | | | | | | | | | |
| The human rights of First Nations Australians are meaningfully considered | | | - | **67** | | **28** | | - | | 6 |  |
| The human rights of First Nations Australians are protected | | | - | **78** | | **17** | | - | | 6 |  |
| There is improvement of First Nations Australian individuals' and communities' lives | | | - | **56** | | **28** | | 6 | | 11 |  |
| The process is informed by the priorities and needs of First Nations Australian community/ies that are affected/impacted | | | - | **72** | | **22** | | - | | 6 |  |
| The process is driven and directed by First Nations Australians’ leadership and governance | | | 11 | **50** | | **33** | | - | | 6 |  |
| First Nations Australians have significant influence and power over the process | | | - | **56** | | **33** | | - | | 11 |  |
| The process is informed by the priorities and needs of First Nations Australian community/ies that are affected/impacted | | | - | **72** | | **22** | | - | | 6 |  |
| The diversity of First Nations Australians is recognised and accepted | | | - | **67** | | **28** | | - | | 6 |  |
| Round 2 | | | | | | | | | | | |
|  | | | 1–2^b^ | | 3–5^c^ | | 6–7^d^ | | No Response | | |
| *Q3 Self-determination in alcohol policy, requires the policy makers to use processes that ensure First Nations Australians* | | | | | | | | | | | |
| communities can control the policy-making process throughout | | | 6 | | 39 | | 56 | | - | | |
| communities define the policy-making process | | | - | | 39 | | 61 | | - | | |
| are involved in parts of the policy-making process | | | - | | 28 | | 67 | | 6 | | |
| community/ies have autonomy in the policy-making process | | | - | | 33 | | 67 | | - | | |
| local culture and language/s are considered and adjusted for in the policy-making process | | | - | | 17 | | **83** | | - | | |
| are involved in evaluating the policy | | | 6 | | 11 | | **83** | | - | | |
| are involved in monitoring the policy | | | - | | 17 | | **83** | | - | | |
| are resourced and funded to be included at all stages | | | - | | 17 | | **83** | | - | | |
| communities be able to hold the policy makers accountable | | | - | | 11 | | **89** | | - | | |
| & policy makers can develop and build trust throughout | | | - | | 11 | | **89** | | - | | |
| are involved in throughout the policy-making process | | | - | | 11 | | **89** | | - | | |
| have the opportunity to contribute to the policy-making process | | | - | | - | | **89** | | 11 | | |
| are consulted early in the policy-making process | | | - | | - | | **94** | | 6 | | |
| receive feedback promptly and in a suitable format | | | - | | - | | **100** | | - | | |
| are given adequate time for decision making | | | - | | - | | **100** | | - | | |
| are involved in the co-design/ co-development of policy | | | - | | - | | **100** | | - | | |
| Round 3 | | | | | | | | | | | |
| *Q3 Self-determination in alcohol policy, requires the policy makers to use processes that ensure First Nations Australians* | | | | | | | | | | | |
| communities can control the policy-making process throughout | | | 6 | | 41 | | 53 | | - | | |
| communities define the policy-making process | | | 6 | | 24 | | 71 | | - | | |
| are involved in parts of the policy-making process | | | - | | 6 | | **94** | | - | | |
| community/ies have autonomy in the policy-making process | | | - | | 29 | | 71 | | - | | |
| are involved in ALL data processes relating to alcohol policy (data sovereignty) | | | - | | 24 | | 76 | | - | | |
| two-way sharing (decision-making power and being informed of what has worked elsewhere) | | | - | | 18 | | **82** | | - | | |
| *Q4 Self-determination in alcohol policy development, requires decision-making processes that... Please rank the following statements by the importance of it for self-determination-led alcohol policy development* | | | | | | | | | | | |
| Round 2 | | | | | | | | | | | |
|  | | | 1–2^b^ | | 3–5^c^ | | 6–7^d^ | | No Response | | |
| *Q4 Self-determination in alcohol policy development, requires decision-making processes that... Please rank the following statements by the importance of it for self-determination-led alcohol policy development* | | | | | | | | | | | |
| gives First Nations Australian communities/ participants veto power at all levels | | | 22 | | 44 | | 33 | | - | | |
| are consensus-based | | | 6 | | 44 | | 50 | | - | | |
| are democratic | | | - | | 39 | | 61 | | - | | |
| is defined by First Nations Australians | | | - | | 17 | | **83** | | - | | |
| is led by First Nations Australians | | | - | | 11 | | **89** | | - | | |
| are adapted for local context | | | - | | 6 | | **94** | | - | | |
| are evaluated and monitored, with prompt response to feedback | | | - | | 6 | | **94** | | - | | |
| recognises the cultural obligations and expectations of First Nations Australians | | | - | | 6 | | **94** | | - | | |
| are participatory and transparent for all parties | | | - | | - | | **100** | | - | | |
| involves First Nations Australians | | | - | | - | | **100** | | - | | |
| Round 3 | | | | | | | | | | | |
| *Q4 Self-determination in alcohol policy development, requires decision-making processes that... Please rank the following statements by the importance of it for self-determination-led alcohol policy development* | | | | | | | | | | | |
| gives First Nations Australian communities veto power at all levels | | | 6 | | 47 | | 47 | | - | | |
| are consensus-based | | | - | | 35 | | 65 | | - | | |
| are democratic | | | - | | 24 | | 76 | | - | | |
| gives First Nations Australian community controlled organisations the collective veto power at all levels | | | 18 | | 29 | | 53 | | - | | |
| are balanced between the evidence-base and community preferences | | | - | | 29 | | 71 | | - | | |
| are not circumvented or changed at higher tiers of government | | | - | | 24 | | 76 | | - | | |
| Round 2 | | | | | | | | | | | |
|  | | | 1–2^b^ | | 3–5^c^ | | 6–7^d^ | | No Response | | |
| *Q6 At implementation, alcohol policy should include approaches that ensures it...* | | | | | | | | | | | |
| is translatable across the wider government and policy systems | | | 6 | | 28 | | 67 | | - | | |
| supports First Nations Australian leading service provision | | | - | | 22 | | 78 | | - | | |
| results in the changes desired by the affected community/ies | | | - | | 17 | | **83** | | - | | |
| involves First Nations Australians in the resource allocation decision-making | | | - | | 17 | | **83** | | - | | |
| involves First Nations Australians in the implementation decision-making | | | - | | 6 | | **94** | | - | | |
| is evaluated and monitored, with prompt response to feedback | | | - | | - | | **100** | | - | | |
| is not discriminatory against First Nations Australians human rights | | | - | | - | | **100** | | - | | |
| is respectful of the priorities of First Nations Australians and their communities | | | - | | - | | **100** | | - | | |
| Round 3 | | | | | | | | | | | |
| *Q6 At implementation, alcohol policy should include approaches that ensures it...* | | | | | | | | | | | |
| is translatable across the wider government and policy systems | | | 6 | | 29 | | 65 | | - | | |
| supports First Nations Australians leading service provision | | | - | | 24 | | 76 | | - | | |
|  | | |  | |  | |  | |  | | |
| *a* | *Results for Question 5 are included in the main manuscript* | | | | | | | | | | |
| *b* | *1–2:* | *not self-determination* | | | | | | | | | |
| *c* | *3–5:* | *possibly* | | | | | | | | | |
| *d* | *6–7:* | *definitely self-determination* | | | | | | | | | |
