# Supplementary material for: First Nations Australians’ self-determination in health and alcohol policy development: a Delphi study
Source: Health Res Policy Syst. 2022 Jan 21;20:12. doi: 10.1186/s12961-022-00813-6 (PMC8777453; doi:10.1186/s12961-022-00813-6)

Additional Figure 2: Ranking of values that should facilitate First Nations Australians' self-determination (Q2)

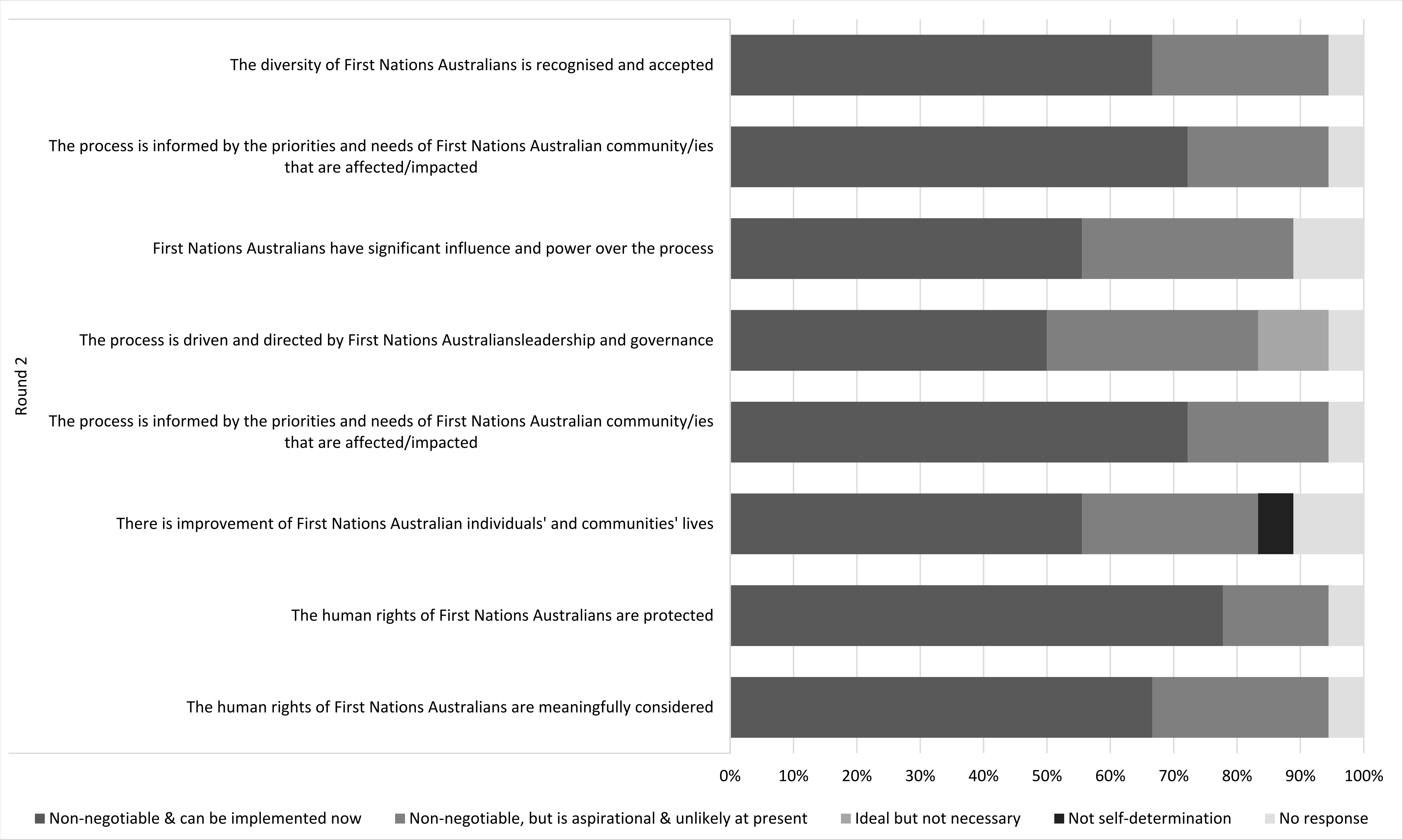

Supplement: Supplementary file 3 — Additional file 3: Figure S2. Ranking of values that should facilitate First Nations Australians' self-determination (Q2). Presents the rankings by proportion for all responses in Q2 for rounds 2 and 3. [file 12961_2022_813_MOESM3_ESM.pdf]
