# Supplementary material for: First Nations Australians’ self-determination in health and alcohol policy development: a Delphi study
Source: Health Res Policy Syst. 2022 Jan 21;20:12. doi: 10.1186/s12961-022-00813-6 (PMC8777453; doi:10.1186/s12961-022-00813-6)

Additional Figure 4: Decision-making processes needed policy development for self-determination (Q4)

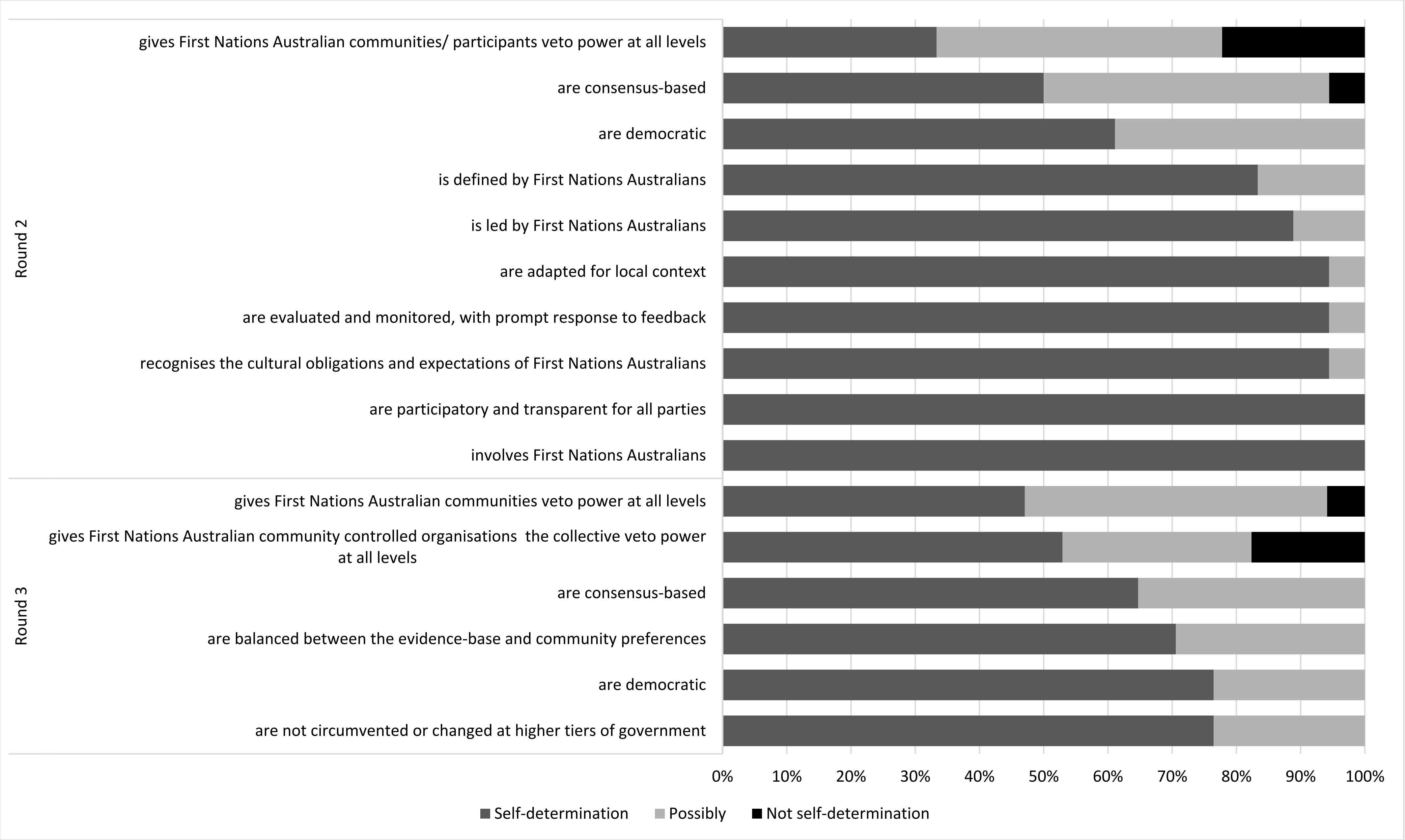

Supplement: Supplementary file 5 — Additional file 5: Figure S4. Decision-making processes needed policy development for self-determination (Q4). Presents the rankings by proportion for all responses in Q4 for rounds 2 and 3. [file 12961_2022_813_MOESM5_ESM.pdf]
