# Supplementary figures and images for: First Nations Australians’ self-determination in health and alcohol policy development: a Delphi study
Source: Health Res Policy Syst. 2022 Jan 21;20:12. doi: 10.1186/s12961-022-00813-6 (PMC8777453; doi:10.1186/s12961-022-00813-6)

Additional Figure 5: Factors necessary for self-determination in the implementation (Q6)

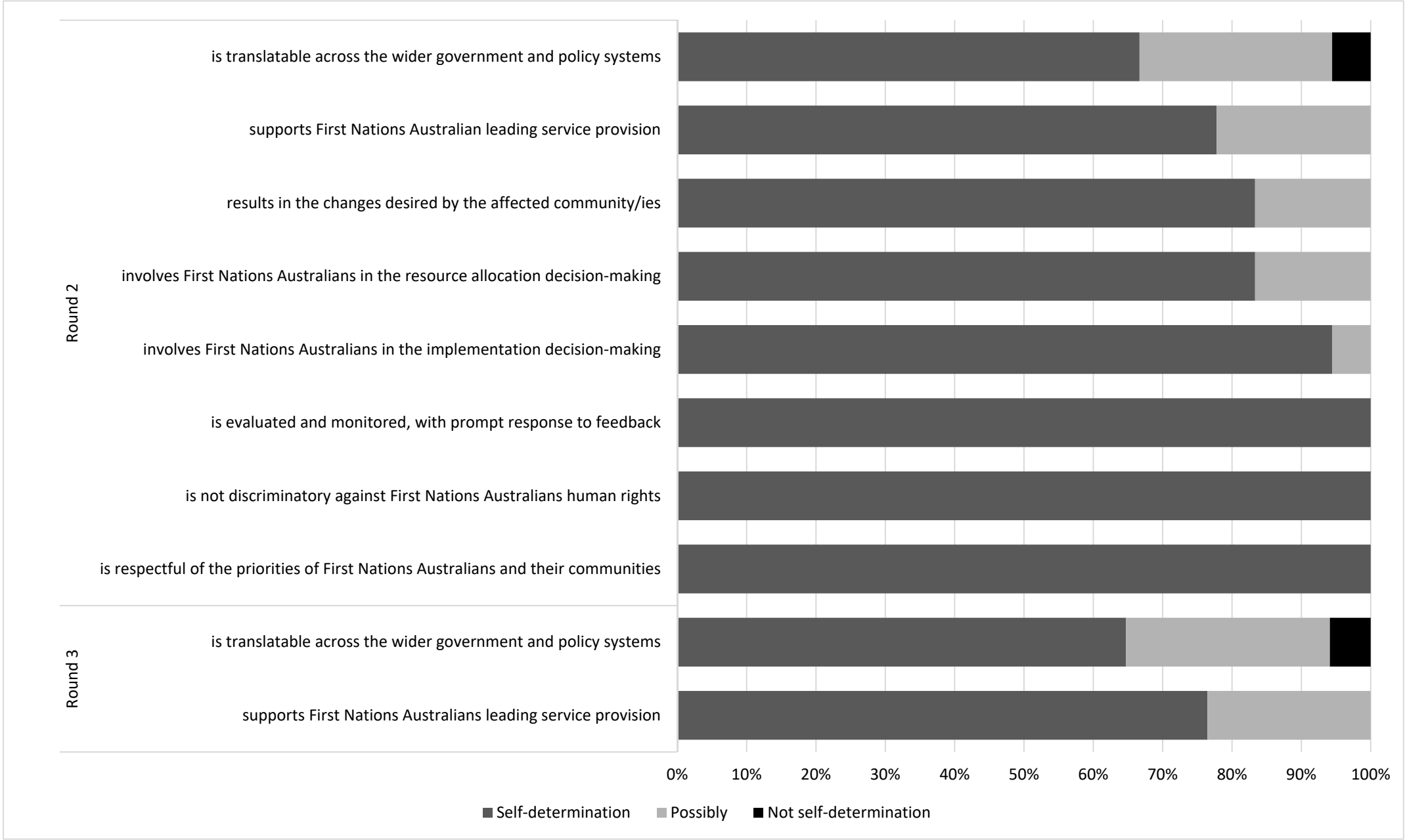

Supplement: Supplementary file 6 — Additional file 6: Figure S5. Factors necessary for self-determination in the implementation (Q6). Presents the rankings by proportion for all responses in Q6 for rounds 2 and 3. [file 12961_2022_813_MOESM6_ESM.pdf]
